# Supplementary material for: Increased Invasion Risk of Tagetes minuta L. in China under Climate Change: A Study of the Potential Geographical Distributions
Source: Plants (Basel). 2022 Nov 26;11(23):3248. doi: 10.3390/plants11233248 (PMC9737557; doi:10.3390/plants11233248)
Supplement: Supplementary file 1 [file plants-11-03248-s001.zip › plants-1967754-supplementary.pdf]

Supplementary material

Variable	Description	In the model (YES/NO)	Unit
Bio1	Annual mean temperature	NO	°C
Bio2	Mean diurnal range	YES	°C
Bio3	Isothermality	YES	-
Bio4	Temperature seasonality	NO	°C
Bio5	Max temperature of the warmest month	NO	°C
Bio6	Min temperature of the coldest month	NO	°C
Bio7	Temperature annual range	NO	°C
Bio8	Mean temperature of the wettest quarter	YES	°C
Bio9	Mean temperature of the driest quarter	NO	°C
Bio10	Mean temperature of the warmest quarter	NO	°C
Bio11	Mean temperature of the coldest quarter	NO	°C
Bio12	Annual precipitation	YES	mm
Bio13	Precipitation of the wettest month	NO	mm
Bio14	Precipitation of the driest month	NO	mm
Bio15	Precipitation seasonality	YES	-
Bio16	Precipitation of the wettest quarter	NO	mm
Bio17	Precipitation of the driest quarter	YES	mm
Bio18	Precipitation of the warmest quarter	YES	mm
Bio19	Precipitation of the coldest quarter	YES	mm
Altitude	Altitude	YES	m
T_Gravel	Topsoil gravel content	YES	%vol.
T_Sand	Topsoil sand fraction	YES	%wt.
T_Silt	Topsoil silt fraction	NO	%wt.
T_Clay	Topsoil clay fraction	YES	%wt.
T_OC	Topsoil organic carbon	YES	% weight
T_pH_H2O	Topsoil pH(H2O)	NO	-
T_BS	Topsoil base saturation	YES	%
T_TEB	Topsoil TEB	NO	cmol /kg
T_CaCO3	Topsoil Calcium Carbonate	NO	% weight
T_CaSO4	Topsoil Gypsum	NO	% weight
T_ESP	Topsoil sodicity(ESP)	NO	%
T_ECE	Topsoil salinity(Elco)	YES	dS/m

Figure S1. Environmental variables related to the distribution of *Tagetes minuta* L.

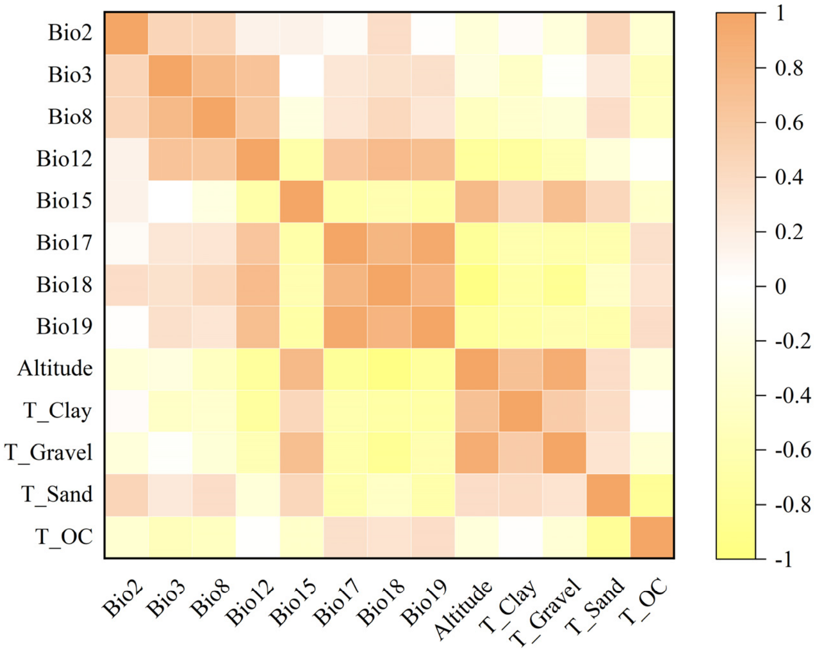

Figure S2. Correlation coefficient between Environmental variables.
